# Attachment and parental reflective functioning features in ADHD: enhancing the knowledge on parenting characteristics

**DOI:** 10.3389/fpsyg.2015.01313

**Published:** 2015-09-01

**Authors:** Clarissa Cavallina, Chiara Pazzagli, Veronica Ghiglieri, Claudia Mazzeschi

**Affiliations:** ^1^Department of Philosophy, Social, Human and Educational Sciences, University of PerugiaPerugia, Italy; ^2^Sezione di Neuroscienze Sperimentali, Fondazione Santa Lucia IRCCSRome, Italy

**Keywords:** parenting, ADHD, risk factor, attachment, reflective functioning

## Abstract

Attention-Deficit/Hyperactivity Disorder (ADHD) is a disorder characterized by a chronic, pervasive, and developmentally inappropriate levels of impulsivity and in attention. It is associated with adverse academic and social functions and stress to families. Studies provide evidence that family variables are correlated with this disorder and that parenting styles play an important role in its complexity. However, a thorough investigation of the impact of parental affective and relational aspects on the ADHD child’s areas of functioning is still needed. In designing future research on ADHD, we suggest to investigate parenting characteristics to a greater extent by adopting an attachment perspective with a focus on parental reflective functioning as it pertains to the child’s ADHD clinical condition.

## Introduction

Attention-Deficit/Hyperactivity Disorder (ADHD) is a neurodevelopmental disorder with characteristics of inattention, hyperactivity, and impulsivity. It shows great heterogeneity in symptoms and functioning (Wahlstedt et al., 2009). Beyond the core symptoms of ADHD, functional impairment in multiple contexts is integral to the diagnosis. Children with similar ADHD symptoms exhibit varying degrees of impairment across social, academic, and family contexts. It is now proven that hereditary factors are important in the etiology of ADHD and that neurobiological aspects are crucial in its definition (Thapar et al., 2013). Nevertheless, valuable longitudinal studies demonstrate how both genetic and environmental variables are intimately related and interact with each other, thereby influencing the expression of ADHD. In the last two decades, more attention has been paid to the investigation of environmental aspects playing a crucial role in ADHD. Strong correlations have been found between parenting and ADHD in children.

The diverse definitions of parenting can be organized into two dimensions. One pertains to parenting in terms of behaviors, practices or styles, and the other relates to attachment (Cummings and Cummings, 2002). According to previous studies, attachment has a quality that transcends the day-to-day interactions between parent and child. It is considered to be highly significant in the study of parenting. Findings on the association between parenting factors and occurrence of ADHD symptoms are particularly important because it is generally assumed that there is considerable interaction between psychosocial influences and children’s impairment in key areas of behavioral, cognitive, and psychosocial functioning (Bornstein, 2002). Moreover, considering that ADHD clinical condition can be extremely variable, it has been evidenced that parenting influences are crucial for understanding the factors that contribute to the behavioral expression and different developmental trajectories of ADHD (Sonuga-Barke and Halperin, 2010).

One aspect that has not yet been clearly elucidated is the role of the parent’s attachment characteristics and specifically of parental reflective functioning. This characteristic is the parent’s cognitive capacity in relation to attachment (Fonagy et al., 2002). The aim of this perspective is to raise awareness of the importance for investigating the influence of parent’s attachment patterns and of parental reflective functioning. Parental reflective functioning is the parents’ capacity to reflect upon their own and their child’s internal mental experience. It is necessary for the development of cognitive abilities in the child and for promoting affect regulation and productive social relationships, which are impaired in ADHD children (Nijmeijer et al., 2008).

## ADHD and Parenting Characteristics

The maternal hostility of non-biological (adoptive) mothers toward children with a biological predisposition for ADHD is predictive of an ADHD diagnosis (Harold et al., 2013). This concept emphasizes the impact of relational aspects on the development of ADHD. Other studies investigated the link between child ADHD with certain parenting characteristics such as higher levels of stress (Theule et al., 2010), marital discord (Wymbs et al., 2008), psychiatric disorders (Chronis et al., 2003), and authoritarian parenting style (Alizadeh et al., 2007). Specific parenting characteristics highly influence child ADHD’s clinical severity, psychiatric comorbidity, and impaired functioning. For example, it has been shown that parenting styles significantly moderate the association of ADHD with other psychiatric symptoms. Specifically, a maternal overprotective parenting style interacts with ADHD symptoms to predict higher anxiety, depression, conduct disorder (CD), antisocial, and borderline symptoms, whereas paternal affection and warmth interact with ADHD symptoms to predict lower anxiety, reduced CDs, and fewer antisocial symptoms (Ni and Gau, 2015). Paternal warmth is also associated with greater peer acceptance and correlates negatively with peer rejection and problematic social behavior (Hurt et al., 2007).

A child’s ADHD challenging behavior can reasonably have an impact on parent behavior and adjustment. Likewise, a parenting style may exacerbate the course of child’s disorder. Over time, both child and parent may be reinforced for maladaptive behaviors, which strengthens the coercive style of relating. In this respect Taylor (1999) argues that ADHD symptomatology leads to critical expressed emotion (EE) from parents and inefficient coping strategies, which in turn contribute to the development of Oppositional Defiant Disorder (ODD). High levels of parental EE is a stressor for ADHD that leads to a larger cortisol response (Christiansen et al., 2010). This means that the development of oppositional behavior problems might be mediated by the stress-response to high EE. Also from the side of treatment there is evidence of this connection. For example, Schachar et al. (1987), investigating the effect of MPH treatment on overactivity and/or defiance, found an improvement of maternal warmth and a reduction of maternal criticism (a component of EE) in families of children who responded to methylphenidate.

Deault (2010) critically reviewed research studies concerning family characteristics associated with the development of internalizing and externalizing psychiatric comorbidities and functional impairments in children with ADHD. Interestingly, the risk and protective factors within the families of children with ADHD were emphasized. Moreover, new research directions were given for deepening our understanding of parenting characteristics that will help to trace the possible developmental pathways in such an extremely wide diversity of the disorder (Deault, 2010). It is clear that ADHD diagnosis alone is not predictive of the child’s negative outcome highlighting the general importance of parent–child interactions on ADHD clinical condition (Gordon et al., 2006). In line with these assumptions, ADHD children, whose parents were highly critical and lacked warmth, had significantly higher levels of ODD and CD (Sonuga-Barke et al., 2009). In addition, with regard to the development of internalizing symptomatology, it has been found that an inconsistent parenting style was associated with a deteriorated ADHD clinical condition characterized by depressive symptoms. However, both parent management and locus of control were found to mediate the connection (Ostrander and Herman, 2006). Moreover, maternal anxiety and specific parenting practices such as over-protectiveness and lack of positive parenting, were associated with children’s anxiety symptoms (Pfiffner and McBurnett, 2006).

These findings support the exploration of the impact of specific parenting characteristics on shaping the child’s ADHD trajectory. Parenting includes many types of rearing behavior to study (Bornstein, 2002). Many different parenting variables have been shown to be predictors of ADHD (Johnston and Mash, 2001; Modesto-Lowe et al., 2008; Deault, 2010). However, to our knowledge there is no research on parental reflective functioning and its effect on the expression and development of ADHD.

## Experimental Findings on ADHD in Translational Neuroscience

Recent experiments in animal models of disease (Francis et al., 1999; Champagne et al., 2003; Weaver et al., 2004) show the significant relevance of environmental factors. They suggest that both maternal and paternal experiences alter the development in their offspring (Curley et al., 2011a,b). However, in all mammalian species, there is an intense early period of maternal influence that is more significant than the paternal one, demonstrating the mother–baby relationship is exclusive and greatly influential in the development of the offspring (Champagne et al., 2003).

In the last decade, numerous behavioral studies conducted on laboratory animals clearly demonstrated that maternal care plays a role in the epigenetic programming of genes function during early life (Weaver et al., 2004). Most of these studies have explored the effects of early life environment on stress programming through variations in the quality of early postnatal maternal care, including specific parenting behaviors. The results clearly support the view that mothering requires attentional commands to focus on infant and respond contingently to his needs. A mother must also have the cognitive capacity to switch her attention efficiently across many social and situational demands in highly stimulating environments while maintaining and manipulating information in her working memory to plan and guide mother–infant interactions (Barrett and Fleming, 2011). This critically important early social experience will constitute the basis of empathy and pro-social behavior in adult subjects. Similarly, the integrity of maternal behavior is linked to executive functions such as attentional set-shifting and prepulse inhibition (Lovic and Fleming, 2004; Afonso et al., 2007). Furthermore, it has been shown that rats’ maternal deprivation is related to deficits in these executive function-processes (Lovic and Fleming, 2004; Burton et al., 2006; Garner et al., 2007).

Clinical studies for identifying biomarkers for developmental disorders in human patients are difficult to conduct because of ethical issues, lack of proper experimental control over the subject’s environment and genetic background, and inaccessibility of brain tissue for analysis. In this regard, animal models are helpful and provide a complementary approach for understanding the processes by which maternal behavior during pregnancy and postpartum period influences the physiology and the psychosocial behavior of offspring. Aberrant maternal behavior during this period of development can result in the development of behavioral and emotional disorders that may persist into adulthood. Sterley and co-workers showed that life stress increases the risk of developing a psychiatric disorder later in life, possibly by altering specific neural networks that have also been implicated in ADHD (Sterley et al., 2013). In particular, in a validated rat model of ADHD, the spontaneously hypertensive rat, the characteristic hyperactive behavior was enhanced following maternal separation, and abnormally low levels of anxiety-like behavior were reduced even further, as measured by behavioral tests (Sterley et al., 2011).

The influence of maternal care on epigenetic modifications, gene expression, and neuroendocrine functions in offspring is complex. From an evolutionary point of view the impact of maternal behavior is critical since maternal care styles are transmitted across generations as an early model of social behavior. Therefore, although significant advances in understanding the origin of individual differences in behavior have been made, there are still many open questions on the link between maternal care and altered gene activity that should be explored using a multidisciplinary approach across animal species, when investigating on the causes of a disease.

## ADHD, Attachment and Parental Reflective Functioning

Attachment theory can be conceived as an affect regulation theory (Mikulincer et al., 2003). According to this perspective, children with insecure attachment, who have difficulties in self-regulation, encounter difficulties in controlling their impulses in multiple contexts throughout their lives (Belsky, 1999). It has been observed that characteristics of insecure attachment resemble those of ADHD, including difficulties in emotional and behavioral regulation, such as impulse control, self-calming, persistence, and patience, as well as social difficulties (Clarke et al., 2002).

In the limited literature on attachment and ADHD, a predictive link between the two has been empirically observed. Specifically, a recent review has emphasized an association between ADHD and a lack of attachment competences (Storebø et al., 2013). Two main types of studies are identified in the field of attachment and ADHD. One type of studies is focused on attachment in ADHD diagnosed subjects. A second type of studies focuses on attachment in parents of ADHD siblings. Most of these studies were conducted by measuring the attachment patterns in adolescents or children with ADHD. There are only a few papers that explore parents’ attachment characteristics and ADHD siblings.

Regarding the attachment in ADHD children it has been found that insecure attachment representation in adolescents is significantly related to an ADHD clinical condition characterized by major impairments in attentive abilities and impulsive regulation compared with securely attached adolescents (Guarino et al., 2012). Similarly, Scharf et al. (2014) suggested that insecure attachment styles in adolescence may serve as developmental precursors for ADHD symptomatology, rejection sensitivity, and social adjustment. Moreover, Scholtens et al. (2014) investigated ADHD symptoms in relation to attachment representations in children by using both attachment and non-attachment-related story stems. They found that insecurely attached children responded to non-attachment related story stems mostly incoherently and negatively. Specifically, insecure disorganized children received significantly higher ratings of ADHD symptoms, and their narratives had higher levels of negative content than did children classified as organized. There were no significant effects of attachment on conduct problems, vocabulary, disinhibition, sustained attention, or working memory deficits. Storebø et al. (2014) argue that treatment for ADHD must incorporate new social skills training which can help the children to deal with their attachment problems. Regarding the second type of studies, papers on parent’s attachment styles and representations are scarce. Two case studies examined attachment and ADHD. One case study researched family dynamics and attachment strategies in a family with ADHD (Dallos and Smart, 2011). A second case study explored the possible effects of the insecure disoriented attachment patterns of a child and of his mother with respect to ADHD (Crittenden and Rindal Kulbotten, 2007). The study of Kissgen et al. (2009) showed that maternal insecure attachment is associated with higher ADHD symptom load. Furthermore, maternal (but not paternal) attachment insecurity was found to be significantly associated with the severity of a clinical sample of toddlers’ emotional and behavioral problems, such as hyperactivity and irritability (Karebekiroğlu and Rodopman-Arman, 2010). These findings suggest that studies on patterns of attachment of parents should be increased to better understand its relation with the ADHD child’s clinical condition. In general, these findings suggest that children’s and parent’s attachment characteristics may play a role in the modulation of the clinical expression of the child’s ADHD symptomatology and of its related impairments. Conway et al. (2011) showed that ADHD children experience higher incidences of chronic stress (environmental trauma) and greater disruptions in early attachment relations (attachment trauma) compared with non-ADHD counterparts. Moreover, similar to the observations in animal studies, it has been shown that early attachment deprivation predicted a worsening of ADHD symptoms in a later stage (Roskam et al., 2014). Elevated rates of attention deficit and overactivity have been noted in samples of institutionally reared children. There is some evidence that inattention and overactivity symptoms might constitute a specific deprivation syndrome in children with severe early deprivation when it is linked with attachment difficulties. Furthermore, it is not explicable as a secondary consequence of a cognitive deficit or on the basis of malnutrition (Kreppner et al., 2001). It has been suggested that this high comorbidity of attachment trauma with ADHD diagnosis or an ADHD-like symptomatology could indicate a possible impairment of mentalization during child’s development (Conway et al., 2011). In fact, cognitive capacities seem to develop through early attachment relationships that create the opportunity for the child to learn about mental states and determine the depth to which the social environment can ultimately be processed (Fonagy et al., 2002). In that context, parental reflective functioning, the parent’s capacity to reflect upon her own and her child’s internal mental experience, promotes cognitive abilities in the child and seems to be an important aspect of parenting connected to attachment.

As children develop mentalizing capacities, they learn to adapt to different situations and be flexible in their responses. This ability to understand and interpret themselves and others within their environment “affects regulation, impulse control, self-monitoring, and the experience of self agency” (Fonagy and Target, 1998). It is interesting to note that all of the above-mentioned skills have been found to be impaired in children with ADHD (Conway et al., 2011). Nevertheless, the studies on parenting and attachment factors linked to ADHD have not extensively investigated the role of parental reflecting functioning relevant to ADHD. In addition, although ADHD is clearly associated with impairment in the social domain, studies investigating social cognition processes in ADHD and parenting aspects that impact on these abilities in ADHD children are inexplicably scarce. ADHD is associated with social cognition impairments, involving emotional face and prosody perception and there is also some evidence of theory of mind deficit and reduced empathy in ADHD subjects (Uekermann et al., 2010). In this context, we advise the unmet need of widening our knowledge of the impact of parental reflective functioning in ADHD children, in relation to attachment characteristics.

## Conclusion

Although it is generally accepted that child ADHD is linked to specific parenting characteristics, parental attachment patterns should be analyzed to a greater extent. Several studies have evidenced an association between insecure attachment characteristics in children and adolescence with ADHD and their clinical condition. However, to our knowledge, little research is available regarding the exploration of the parent’s attachment patterns, although intergenerational transmission of attachment patterns has been clearly established. This scarcity of research highlights the importance of deepening our understanding on mother’s and father’s attachment characteristics with their parental reflective functioning in relation to their child’s ADHD clinical condition.

A core element of parent’s reflective functioning involves their capacity to reflect upon the child’s uniquely subjective intentions during moments of stress and conflict, opening the child’s possibility to develop affect modulation (Fonagy et al., 2002; Slade, 2005). Moreover, parental reflective functioning allows the caregivers to contain the child’s distress, giving rise to mutual regulatory processes that gradually increases the child’s ability to self-regulate (Grienenberger et al., 2005). Alterations in this capability could possibly be a predictor of the quality of parent-child relation and a key factor in conceptualizing the risk and protective variables that shape ADHD pathways. We envision that by adopting an attachment perspective, this field of research may open new avenues for translational studies aimed at clarifying the psychobiological link between parenting characteristics and ADHD and it would promote the design of new therapeutic approaches for personalized interventions.

## Conflict of Interest Statement

Conflict of Interest Statement: The authors declare that the research was conducted in the absence of any commercial or financial relationships that could be construed as a potential conflict of interest.
